# *Clostridium Difficile* Infection Worsen Outcome of Hospitalized Patients with Inflammatory Bowel Disease

**DOI:** 10.1038/srep29791

**Published:** 2016-07-15

**Authors:** Ting Zhang, Qian-Yun Lin, Jia-Xi Fei, Yan Zhang, Min-Yi Lin, Shuang-Hong Jiang, Pu Wang, Ye Chen

**Affiliations:** 1 Department of Gastroenterology, State Key Laboratory of Organ Failure Research, Nanfang Hospital, Southern Medical University, Guangzhou 510515, China; 2 Infection Disease Center, the Fifth Affiliated Hospital Sun Yat-Sen University, Zhuhai, 519000, China

## Abstract

The prevalence of *Clostridium difficile* infection (CDI) in patients suffering from inflammatory bowel disease (IBD) has increased rapidly over the past several decades in North America and Europe. However, the exact global epidemiology remains unclear because of insufficient data from developing countries. A total of 646 hospitalized adult IBD patients were enrolled; and their fresh stool specimens were obtained and used for *Clostridium difficile* detection. The incidence of CDI in Crohn’s disease (CD) patients (12.7%) was significantly lower than that in Ulcerative disease (UC) patients (19.3%). Among the toxin types, A^+^B^+^ strain was the most common. Length of stay, hospitalization frequency and bowel surgery rate were significantly higher in the CDI than in the non-CDI group in CD or UC patients. More patients in CDI-CD group were still in active and even clinical moderate or severe CD stage than non-CDI-CD group after 2 years of following-up. Fistula, antibiotics and infliximab usage likely increased the CDI rate in CD patients, Infliximab treatment was considered a risk factor in UC patients. CDI is an exacerbating public health issue that may influence IBD course, increase expenditures, and delay the remission of IBD patients. IBD patients with CDI require urgent attention.

Inflammatory bowel disease (IBD) is characterized by chronic relapsing inflammatory conditions. This disease frequently requires long-term medical therapy, periodic hospitalization, and surgery. Intestinal microbiota is reported to play an important role in the onset and progression of IB^1^. Among microbiota, *Clostridium difficile*, a Gram-positive anaerobic spore-forming bacterium, causes various diseases, including asymptomatic carriage, mild diarrhea, colitis, or pseudomembranous colitis. Many patient subgroups, especially the elderly, immunocompromised individuals, oncology patients, and IBD patients, are possibly at increased risk of *C. difficile* infection (CDI)^2^,^3^. CDI may be difficult to distinguish from an IBD flare and may contribute to the exacerbation and relapse of IBD. Endoscopy rarely reveals pseudomembranes and thus unhelpful for CDI diagnosis in IBD^4^. In accordance with the guidelines for the management of IBD in adults (2010), IBD patients with diarrhea should be subjected to microbiological testing for *C. difficile* toxins^5^,^6^. The guidelines for the diagnosis, treatment, and prevention of CDI (2013) also indicate that all patients affected by IBD and hospitalized with a disease flare should undergo testing for CDI^7^.

The prevalence of CDI in IBD patients has increased rapidly over the past several decades^8^,^9^,^10^,^11^. The infection rates vary greatly in different countries or periods. It is reported that approximately 1.4%, 2.3%, and 2.9% of all IBD hospitalizations were complicated by CDI in the United States during 1998, 2004, and 2007, respectively^10^. Patients with IBD and CDI also yield a higher mortality and risk of colectomy^8^,^12^. The increasing prevalence and incidence and exacerbating outcome of CDI in IBD patients have been variably reported in North America and Europe^2^,^9^,^10^,^12^,^13^. However, the exact global epidemiology remains unclear because of insufficient data from developing countries. The lack of regulated antibiotic use in such countries suggests that the prevalence of CDI may be comparatively high. The awareness and surveillance of CDI in Asia have also remained poor^14^. CDI is not considered a notifiable infection and is thus rarely reported in mainland China. The incidence and prevalence of CDI in IBD patients also remain ambiguous in China^15^.

Although epidemiological and microbiological research has indicated that CDI plays an important role in the initiation and exacerbation of IBD, the association between the clinical characteristics of IBD and CDI is unknown^16^. In a general population, the risk factors of CDI include advancing age, severe underlying illness, hospitalization, naso-gastric tube usage, recent or current antibiotic use, anti-neoplastic chemotherapy, and immunosuppressants; the exact relationship between the use of proton pump inhibitors (PPIs) and CDI is also increasingly recognized^17^,^18^. However, this situation seems different from that observed in IBD patients. Few studies on CDI in IBD patients have been published, and available data are mainly from North America and Europe. In early studies, C. difficile is mainly detected through ELISA, which yields a lower positive rate than PCR does. Conducted in British Columbia, a larger-population-based retrospective analysis considered IBD-associated medicinal factors and revealed that the rate of CDI was tripled following corticosteroid initiation(RR = 3.4; 1.9–6.1) compared with other immunosuppressant agents, reached up to 14/1000 (10.6–18.2). However, infliximab is not significantly associated with CDI^19^. In a recent retrospective pediatric study, the risk of CDI was associated with the severity of IBD and increase in patient age^20^. Among the common CDI risk factors in IBD patients, antibiotic use does not seem to play a critical role probably due to the alteration in colonic microbiota in IBD^13^. However, data regarding the CDI prevalence in adults with IBD in China remains to be determined.

Our study aimed to evaluate the effect of CDI on IBD patients and its prevalence, risk factors and toxin gene profiles of *C. difficile* strains isolated from patients with IBD through selective anaerobic culture and direct PCR methods in a large teaching hospital in South China.

## Results

### Incidence of CDI in IBD patients

**Figure 1:**
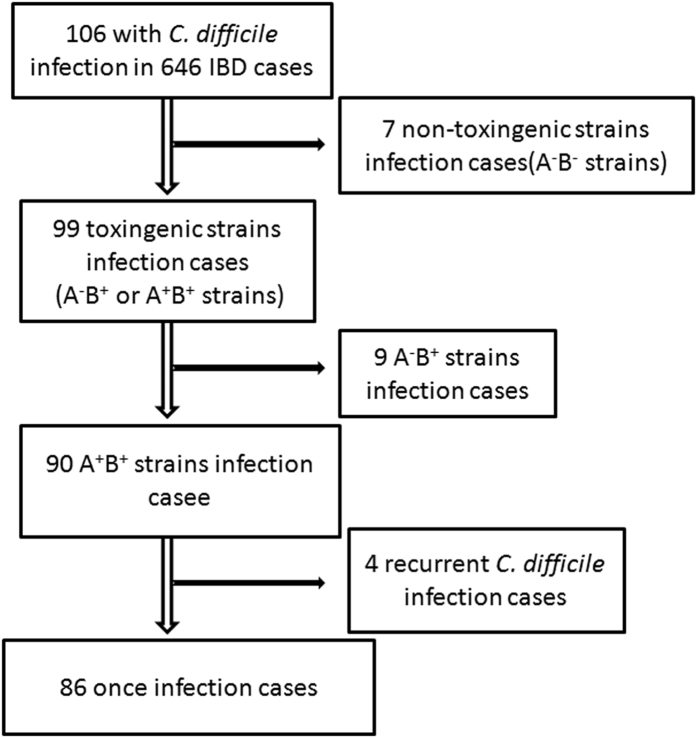
Overview diagram of the *C. difficile* infection cases included in this study.

**Figure 2:**
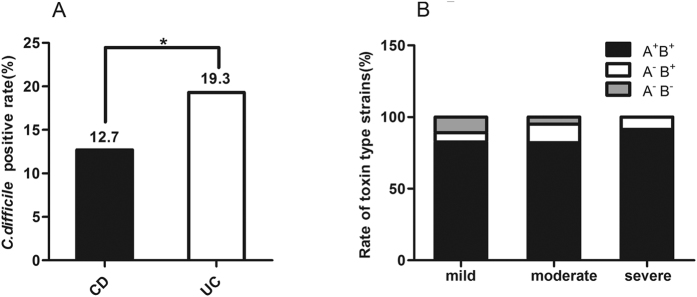
Clinical *C. difficile* infection in IBD patients. (**A**) *C. difficile* positive rate in CD was significantly lower than that in UC patients(12.7% vs 19.3%, *P = 0.022); (**B**) toxin type distribution of *C. difficile* strains according to IBD severity.

A total of 646 IBD patients, including 387 with CD and 259 with UC, were enrolled in our study. Of these patients, 67.2% and 58.7% of male patients were accounted for in CD and UC, respectively. The demographics and clinical characteristics of these patients are listed in Table 1. A total of 106 C. difficile-positive cases, including 7 non-toxigenic (A^−^B^−^) strains, 9 toxin A-negative, B-positive (A^−^B^+^) strains, and 90 toxin A, B-positive (A^+^B^+^) strains, were detected (Fig. 1). Of the A^+^B^+^ toxin type CDI cases, 4 suffered *C. difficile* invasion more than once. Of 387 CD patients, 49 (12.7%) tested positive for toxigenic *C. difficile*(A^+^B^+^ or A^−^B^+^), and this value was significantly lower than 50 (19.3%) in 259 UC patients (*P* = 0.022, Table 1 and Fig. 2A).

### Toxin type of C. difficile

The toxin type distribution of *C. difficile* was classified on the basis of the clinical severity of IBD (Fig. 2B). In each severity level, A^+^B^+^ strain was the most common toxin, and the rates in the mild, moderate, and severe IBD cases were 82.2%, 84.2%, and 91.3%, respectively. The A^−^B^+^ strains accounted for 6.7%, 10.5% and 8.7% from mild to severe IBD stage, respectively. However, the A^−^B^−^ strains were detected mainly in mild (11.1%) and moderate (5.3%) cases, but not in severe IBD patients. More importantly, The first hyper-toxigenic epidemic strain named ribotype 027 was isolated from one of these CD patients^21^.

### CDI influence the IBD course

We further analyzed the progression characteristics of CD and UC patients within two years after CDI was detection(Table 2). The length of stay(33 vs 17 median days, *P* < 0.001), hospitalization times(7 vs 3 median times, *P* < 0.001) and bowel surgery rate(38.8% vs 24.9%, OR = 1.92, *P* = 0.042) were significantly higher in CDI-CD patients than in non-CDI–CD patients. Furthermore, 73.5% of the CDI-CD patients did not undergo remission after 2 years, and this value was significantly higher than that in active stage in non-CDI-CD patients (54.1%, OR = 2.35, *P* < 0.05). More CDI–CD patients were still in the active stage (*P* = 0.013) and even in the clinical severe or moderate CD stage (*P* = 0.046) than non-CDI–CD patients after 2 years of follow-up. However, the CDI group with UC patients required longer length of stay, higher frequency of hospitalization, and higher rate of bowel surgery than the non-CDI group did. Nevertheless, this finding did not imply disease activity and severity differences.

### Risk factors of CDI in IBD patients

The effects of CDI-associated risk factors, including age, gender, disease history, fistula, perianal abscess, bowel involvement and medication use, were examined through logistic regression analysis (Table 3). Fistula(OR = 2.48, *P* = 0.007), antibiotics(OR = 5.11, *P* < 0.001), and infliximab(OR = 2.22, *P* = 0.012) usage likely increased the CDI rate in CD patients. When antibiotics and infliximab were used simultaneously, the CDI rate increased by 10.21–fold (P < 0.001). Similar to CD cases, UC cases exhibited an increase in the CDI rate after they were treated with infliximab treatment (OR = 2.60, *P* < 0.001). Although antibiotics alone increased the CDI rate without statistical significance, antibiotics combined with infliximab could also increase the CDI rate by 4.53 times (*P* = 0.021). However, the numberof infliximab courses was not related to CDI rate. Antibiotics subgroup analysis revealed that metronidazole (OR = 2.29, *P* = 0.037) and cephalosporin (OR = 2.27, *P* = 0.017) were independent risk factors of CDI in CD patients. Patients with CD (OR = 7.72, *P* = 0.001) and UC (OR = 5.69, *P* = 0.012) were more vulnerable to CDI when metronidazole and infliximab were used in combination. Cephalosporin used with infliximab could also increase the risk of CDI among CD patients (OR = 7.62, *P* < 0.001). By contrast, disease history, bowel involvement, systemic steroids, PPIs, oral 5-aminosalicylic acid (ASA), and immunosuppressant use did not significantly differ between CDI and non-CDI groups in IBD patients (*P* > 0.05).

## Discussion

In summary, this study provides evidence that (i) the incidence of CDI was significantly higher in patients than in CD patients; (ii) A^+^B^+^ *C. difficile* strains were more common in IBD patients; (iii) CDI may influence IBD course, increase costs, and delay IBD remission, especially in CD patients; (iv) infliximab and antibiotic use and fistula were risk factors of CDI in CD-hospitalized patients. Infliximab use may increase the CDI rate in patients who suffered UC.

The reported CDI rates in IBD patients vary in different studies. In North America, the rate increased from 1.8% in 2004 to 4.6% in 2005^12^. In Canada and the USA, CDI occurred in 0.9–2.2% of admissions because of CD and in 1.8–5.7% because of UC, respectively^2^,^8^. In our study involving hospitalized patients, the CDI rate in Chinese IBD patients was reported for the first time. The CDI rates were 12.7% in CD patients and 19.3% in UC patients. Some studies have reported that CDI is more prevalent in UC patients compared with CD patients^8^,^11^. Similarly, the incidence of CDI in our study was also found significantly higher in UC patients than in CD patients. But a study in the Netherlands reported that the prevalence of *C. difficile* was not significantly different between UC (3.4%) and CD patients (5.9%)^22^. We observed that the incidence of CDI in CD and UC patients in our study was higher than that in the USA and Canada. Moreover, The patients enrolled in this study were Asian. The geographical and ethnic differences in patients and different study times may have contributed to the higher infection rates. These inconsistent infection rates may be obtained because all patients enrolled in our study were in-patients, and not a mixture of in- and out-patients, as observed in other studies. In our study, C. *difficile* was detected through morphological and odor identification by using stool cultures. Toxin A (*tcd*A), B (*tcd*B), triose phosphate isomerase (*tpi*), and 16S RNA genes were observed through PCR, which exhibits a higher sensitivity than ELISA does used in other studies. Furthermore, an increased clinical awareness and frequent testing of *C. difficile* in recent years should also be considered.

*C. difficile* plays a crucial role in the pathogenesis of CDI by releasing enterotoxin A and cytotoxin B. These toxins bind to specific receptors on colonic epithelial cells because of their potent cytotoxic and proinflammatory properties, and enter the intracellular space, leading to a systemic inflammatory response, toxic megacolon, and even perforation. The molecular epidemiology of *C. difficile* strains in Asia was mainly A^−^B^+^ ribotype 017 and ribotype 018 strains^14^. While in a recent epidemiological study conducted in Korea, the A^+^B^+^ strains (84.6%), including non-binary toxin producing strains (77.5%) and binary toxin producing strains (7.1%), were most common; the A^−^B^+^ strains were relatively rare (15.4%)^23^. In a prospective study in the Netherlands, toxinogenic *C. difficile* with both *tcd*A and *tcd*B genes was detected in 31% of *C. difficile*-positive samples^22^. Our data showed that A^+^B^+^ *C. difficile* strains were the most common toxin type in CDI-IBD patients, and this finding was similar to that obtained from Korea and higher than those documented in the Netherlands. Nonpathogenic A^−^B^−^ strains were rare and more common in mild disease type. Both toxins can independently induce infection^24^, and the detection of toxin A and toxin B were considered equally in our study. The different distributions of toxin types may be attributed to regional disparity.

A recent study reported that more than half of the CDI-IBD patients required hospitalization, and the colectomy in a 2-year period was 20%^12^. The mortality among hospitalized IBD patients with CDI is fourfold higher than that of patients without CDI in the United States^11^. In CD inpatients as our study researched, the bowel surgery rate could reach 38.8%, which was higher than that in the previous study. In the United States, CDI amounted to approximately USD 1.2–5.9 billion per year of acute hospital care expenditures^25^,^26^. Our data showed that the length of stay was extended, the frequency of hospitalization increased, and the rate of bowel surgery increased in both CD and UC patients with CDI after 2 years of follow-up. Furthermore, the remission rate of the CDI-CD patients declined, and the CD condition remained serious 2 years after CDI was detected. Therefore, CDI has predominantly affected IBD patients and has negatively influenced the rehabilitation of IBD patients, especially CD patients.

Infliximab is a chimeric IgG4 monoclonal antibody targeting TNF-α, which is approved for the treatment of patients with IBD. This antibody can induce the formation of regulatory macrophages with immunosuppressive properties to inhibit proliferation of activated T cells and produces anti-inflammatory cytokines; thus, infliximab is highly efficacious, especially in CD patients who are unresponsive to conventional therapies^27^,^28^,^29^. However, several reports have indicated that infliximab use was associated with opportunistic bacterial infections, including CDI^30^,^31^. A prospective, observational, multicenter study on patients treated with infliximab in North America demonstrated that the rate of serious infections associated with infliximab was 1.37 infections per 100 patient-years^32^. Despite the effect of macrophages, infliximab is active against monocytes^33^, which indicates that cytokines are important in inhibiting intracellular pathogens in granulomas. Therefore, the association with CDI may be considered as an extension of the normal and intended pharmacologic activity of infliximab. Furthermore, a randomized controlled trial in patients with rheumatoid arthritis revealed that serious infections were 3 times more likely to occur in infliximab-treated patients than in placebo-treated individuals^34^. Antibiotics, as a risk factor, were considered less common in IBD patients than in general control populations^4^. For instance, 61% of patients reported a history of antibiotic use within 2 months of CDI detection. The most commonly used antibiotic was ciprofloxacin, an oral fluoroquinolone^12^. In this study, we evaluated the potential IBD associated risk factors for CDI and found that (i) fistula, antibiotics (especially metronidazole and cephalosporin) and infliximab may increase the prevalence of CDI in CD patients, particularly when antibiotics and infliximab were used simultaneously, the CDI rate can be increased sharply by 10-fold; (ii) the high CDI rate in UC patients may be related to infliximab, but the infection rate can increase by four times when infliximab was combined with antibiotics. Issa *et al*. identified that the use of immune modulators, which not only included infliximab, but also adalimumab, azathioprine, 6-mercaptopurine and methotrexate, was an independent risk factor for CDI in IBD patients^12^. In our study, infliximab was the most frequently used immune modulator (48.3% in CD patients; 14.7% in UC patients). As such, the patients were grouped separately for analysis. The inconsistency may be attributed to the patients included in this study who used more infliximab than in Issa’s study, and this increased use was observed to be associated with CDI. Therefore, increased vigilance is necessary to identify possible CDI in IBD patients with infliximab usage.

Our study has several strengths. Our study filled the gaps of CDI incidence among patients with IBD in China and in Asia. We found that infliximab may be an independent risk factor of CDI in IBD patients. In addition, Glutamate dehydrogenase (GDH) screening, stool culture and gene detection means (*tcd*A, *tcd*B, *tpi* and 16S RNA genes) were employed to diagnose CDI in our study. Thus, further details regarding *C. difficile*, including toxigenic and non-toxigenic toxin types, could be obtained, analyzed, and reported. Finally, 2 years following-up of the included patients confer strength to our findings.

Despite these strengths, a few limitations should be considered. First, our single-center study was conducted in a tertiary hospital, and our sample size was insufficiently large. Recurrent CDI and toxin typing cases could not be subjected to in-depth subgroup analysis because of the small number of positive samples. To minimize the selection bias, we recruited IBD patients with various subtypes and disease phases. Second, data on pre-hospital concomitant medication use may have been limited or recorded incompletely. Third, the route of medication was not uniform and different regimens were prescribed.

The length of stay in the hospital rapidly increased for CDI-related hospitalizations. Poor clinical outcomes and high colectomy rates in concomitant CDI and IBD have caused heavy burden on families, health systems, and payers in the recent years. IBD patients with CDI require urgent attention. Our study provided a basis for the epidemiology and clinical disease management of CDI in IBD.Further studies on concomitant CDI and IBD within health systems, hospitals, and practices should be performed to enhance our understanding of this complication and improve the clinical care and prevention for this vulnerable population.

## Materials and Methods

### Patients

A total of 646 adult patients with IBD hospitalized in the Department of Gastroenterology, Nanfang Hospital (a teaching hospital of Southern Medical University, Guangzhou, China) between January 2010 and January 2014 were enrolled in the current study. The inclusion criteria were as follows: (1) hospitalized IBD patients with UC or CD within the study period; (2) ≥16 years of age; (3) with an increase of at least three stools per day. CD patients with isolated upper gastrointestinal disease only (Montreal classification^6^,^35^ L4) were excluded. Fecal specimens were collected from patients with diarrhea and examined for *C. difficile* toxin. Patients with presumptive *C. difficile* infection in the absence of a positive toxin assay were excluded in this analysis. CD and UC was diagnosed on the basis of clinical signs and symptoms and endoscopic, histological, and radiological results in accordance with guidelines for the management of IBD in adults (2010)^6^. The severities of CD and UC was evaluated on the basis of Harvey and Bradshaw’s simplified Crohn’s Disease Activity Index (CDAI) score^36^ and the modified Truelove and Witts score^37^.

### Data collection

The medical records of consenting patients were reviewed by two independent investigators to obtain demographic information. We reviewed the subsequent disease courses of IBD patients from the hospitalization data to verify disease exacerbation or progression 2 years after *C. difficile* was detected and to determine whether CDI exacerbated IBD. The length of stay, frequency of hospitalization, rate of bowel surgery, stool consistency, active stage, and disease severity were considered as follow-up factors. Bowel surgeries, including sub-total colectomy and ileo-anal pouch procedure, were executed when affected patients were unresponsive to intensive medical therapy, dysplasia or carcinoma was detected, disease was poorly controlled, acutely recurrent chronic episodes of ulcerative colitis were recorded, and rectal stump following previous colectomy was retained^6^. The data related to patients hospitalized only once were obtained via telephone follow-up. Potential risk factors, including treatment with antibiotics, systemic steroids, oral 5-aminosalicylic acid (5-ASA), tumor necrosis factor α (TNF-α) antagonist (inflixmab), immunosuppressant (azathioprine, 6-mercaptopurine, methotrexate) and PPIs were classified as positive or negative, respectively. The antibiotics used in this study were mainly cephalosporin and levofloxacin. A metronidazole enema was used in some patients with colonic involvement. The medications included in this study were commonly used IBD or concomitant symptom-associated drugs, the details of in-hospital and pre-hospital related drug use were recorded during hospitalization and were thus accessible. Data on pre- and in-hospital concomitant medication use 6 months before *C. difficile* detection were recorded. Disease history, perianal abscess, fistula and bowel involvement within 1 year were also included for potential risk factors. CD and UC phenotypes (bowel involvement) were defined in accordance with Montreal classification regarding disease location^6^,^35^. All CD patients were classified into 3 groups: ileal disease with or without disease limitation to the cecum (L1), a disease limited to the colon (L2), an ileal disease with disease of the colon beyond the cecum (L3). And patients with UC were distributed as follows: ulcerative proctitis limited to distal rectum to the rectosigmoid junction (E1), left sided UC limited to a proportion of the colorectum distal to the splenic flexure (E2), extensive UC extends proximal to the splenic flexure (E3).

### Detection of Clostridium difficile

**Figure 3:**
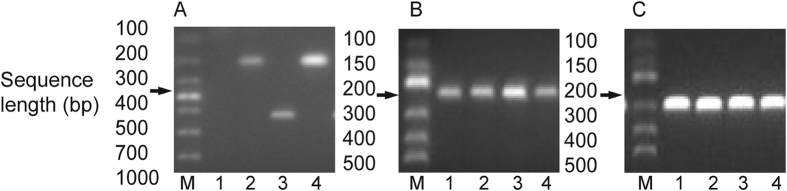
Pictures of the different agarose gels of the PCR products. M, molecular marker; The numbers represent different samples negative or positive for the gene considered and do not refer to the same samples on different gels. (**A**) PCR products of *tcd*A(1, negative sample; 3, positive sample) and *tcd*B(2 and 4, positive samples) gene; (**B**) PCR products of the *tpi* gene indicating the presence of *C. difficile*; (**C**) 16S r RNA gene PCR products.

Fecal specimens were collected from patients with diarrhea and examined for *C. difficile* toxin. The fecal samples were subjected to ethanol shock, cultured on a selective cycloserine cefoxitin fructose agar medium (Oxoid, England, UK), and incubated in an anaerobic jar filled with 10% hydrogen and 10% carbon dioxide (Mart, Netherlands) at 37 °C for 48 h. *C. difficile* colonies were identified on the basis of morphological characteristics and odor. GDH enzyme immunoassay tests (TechLab, VA, USA) were conducted as the first-step screening method. Then, DNAs were extracted from the identified colonies by using a TINAamp bacterial DNA kit (Tiangen, Beijing, China) and utilized to detect toxin A (*tcd*A) and B (*tcd*B) genes through PCR. Triose phosphate isomerase (*tpi*) housekeeping genes^38^,^39^ and 16S RNA genes^40^ were detected to ensure that A^−^B^−^*C. difficile* strains were observed. Therefore, the three main toxigenic types (A^+^B^+^, A^−^B^+^, and A^−^B^−^) of *C. difficile* were distinguished. The specific primers and the corresponding PCR characterizations are shown in Table 4 and Fig. 3, respectively.

### Statistical analysis

Categorical variables were expressed by frequencies and compared through Chi-square analysis. Continuous variables were summarized using median and range and mean and standard deviation. These variables were then analyzed through Student’s *t* test or Wilcoxon Rank Sum test, depending on whether data were normally distributed. Variables were also examined through univariate and multivariate logistic regression to identify the independent risk factors of CD and UC patients for CDI development. OR and confidence interval were calculated for statistically significant factors. All data were analyzed using SPSS 22.0 statistical software (SPSS, Chicago, IL, USA) and a *P* value < 0.05 was considered statistically significant.

### Institutional review board statement

The study was reviewed and approved by the Medical Ethics Committee of the Southern Medical University, Guangzhou, China. The study was conducted according to the principles of the Declaration of Helsinki. All the patients, or their guardians, provided written informed consent before study enrollment.

### Data sharing

The technical support and data set can be obtained at 635464243@qq.com. The presented data are anonymized and the risk of identification is low. No additional data are available.

## Additional Information

**How to cite this article**: Zhang, T. *et al*. *Clostridium Difficile* Infection Worsen Outcome of Hospitalized Patients with Inflammatory Bowel Disease. *Sci. Rep.* **6**, 29791; doi: 10.1038/srep29791 (2016).

## Figures and Tables

**Table 1:** Characteristics of IBD patients (N = 646).

Factors	CD N = 387	UC N = 259	*P*
Age, yrs[Median (range)]	31(16-78)	44(16-75)	<0.001
Male [N (%)]	260(67.2)	152(58.7)	0.028
*C. difficile* positive [N (%)] (%)]	49(12.7)	50(19.3)	0.022
Bowel surgery [N (%)]	103(26.6)	12(4.6)	<0.001
Medication use [N (%)]			
Antibiotics	99(25.6)	120(26.3)	<0.001
metronidazole	44(11.4)	98(37.8)	<0.001
levofloxacin	23(5.9)	38(14.7)	<0.001
cephalosporin	70(18.1)	39(15.1)	0.314
Systemic steroids	162(41.9)	131(50.6)	0.029
Oral 5-ASA	190(49.1)	243(93.8)	<0.001
Immunosuppressant	176(45.5)	38(14.7)	<0.001
Infliximab	187(48.3)	38(14.7)	<0.001
PPI	146(37.7)	78(30.1)	0.046

PPI, proton pump inhibitors.

**Table 2:** Clinical features and outcomes of CD and UC patients with and without toxingenic *C. difficile* infection.

Factors	CD			*P*	UC			*P*
	Positive N = 49	Negative N = 338	OR (95%CI)		Positive N = 50	Negative N = 209	OR (95%CI)	
Length of stay[Median (range),days]	33(3–330)	17(1–268)	1.02 (1.01–1.03)	**<0.001**	21(3–118)	11(1–79)	1.03(1.02–1.05)	**<0.001**
Hospitalization frequency[Median (range)]	7 (1–20)	3 (1–21)	1.28 (1.18–1.39)	**<0.001**	3 (1–12)	1 (1–16)	1.27(1.13–1.42)	**<0.001**
Bowel surgery [N (%)]	19(38.8)	84(24.9)	1.92 (1.03–3.58)	0.042	6(12.0)	6(2.9)	4.61(1.42–14.98)	0.011
Stool consistency [N (%)]								
Formed	22(44.9)	197(58.3)	—		10(20.0)	31(14.8)	—	
Loose, pasty or watery	40(81.6)	301(89.1)	—		16(32.0)	69(33.0)	—	
Bloody mucopurulent	9(18.4)	37(10.9)	—	0.148	34(68.0)	140(67.0)	—	0.451
Active stage [N (%)]	36(73.5)	183(54.1)	2.35 (1.20–4.58)	0.013	45(90.0)	189(90.4)	—	0.926
Severity [N (%)]								
Mild	18(36.7)	172(50.9)	1.0 (refrence)		23(44.9)	97(46.4)	—	
Moderate	21(42.9)	123(36.4)	1.63 (0.83–3.19)		16(32.7)	74(35.4)	—	
Severe	10(20.4)	43(12.7)	2.22 (0.96–5.16)	0.046	11(22.4)	38(18.2)	—	0.632

**Table 3:** Clinical characteristics and potential risk factors associated with toxingenic *C. difficile* infection in CD and UC patients.

Factors	CD			*P*	UC			*P*
	Positive N = 49	Negative N = 338	OR (95%CI)		Positive N = 50	Negative N = 209	OR (95%CI)	
Age, yrs[Median (range)]	31 (16–59)	31(16–78)	—	0.913	45(19–70)	43(16–75)	—	0.797
Male [N (%)]	34(69.4)	226(66.9)	—	0.725	28(56.0)	124(59.3)	—	0.667
History of disease [Median (range),yrs]	3(0.1–19)	2(0.1–43)	—	0.871	2(0.1–20)	3(0.1–30)	—	0.713
Fistula [N (%)]	15(30.6)	51(15.1)	2.48 (1.26–4.88)	0.007	—	—	—	—
Perianal abscess [N (%)]	8(16.3)	33(9.8)	—	0.163	—	—	—	—
Bowel involvement [N (%)]								
L1 or E1	14(28.6)	121(35.8)	—		10(20.0)	45(21.5)	—	
L2 or E2	6(12.2)	45(13.3)	—		17(34.0)	81(38.8)	—	
L3 or E3	29(59.2)	172(50.9)	—	0.268	23(46.0)	83(39.7)	—	0.515
Medication use [N (%)]								
Antibiotics	23(46.9)	76(22.5)	5.11 (2.51–10.39)	**<0.001**	20(40.0)	100(47.8)	—	0.318
metronidazole	10(20.4)	34(10.1)	2.29 (1.05–5.00)	**0.037**	17(34.0)	81(38.8)	—	0.533
levofloxacin	5(10.2)	18(5.3)	–	0.177	5(10.0)	33(15.8)	—	0.299
cephalosporin	15(30.6)	55(16.3)	2.27 (1.16–4.45)	**0.017**	11(22.0)	28(13.4)	—	0.127
Systemic steroids	20(40.8)	142(42.0)	—	0.874	28(56.0)	103(49.3)	—	0.393
Oral 5–ASA	27(55.1)	163(48.2)	—	0.368	45(90.0)	198(94.7)	—	0.211
Immunosuppressant	20(40.8)	156(46.2)	—	0.483	7(14.0)	31(14.8)	—	0.881
Infliximab	32(65.3)	155(45.9)	2.22 (1.19–4.16)	**0.012**	16(32.0)	22(10.5)	2.60 (1.16–5.81)	**<0.001**
Number of infliximab courses	6(1–20)	6(1–17)	—	**0.950**	4(1–12)	6(1–14)	—	0.190
PPI	15(30.6)	131(38.8)	—	0.272	13(26.0)	65(31.1)	—	0.480
Antibiotics^+^ Infliximab	15(30.6)	14(4.1)	10.21 (4.54–22.94)	**<0.001**	5(10.0)	5(2.4)	4.53 (1.26–16.32)	**0.021**
Metronidazole^+^ Infliximab	6(12.2)	6(1.8)	7.72 (2.38–25.01)	**0.001**	5(10.0)	4(1.9)	5.69 (1.47–22.05)	**0.012**
Cephalosporin^+^ Infliximab	10(20.4)	11(3.3)	7.62 (3.04–19.10)	**<0.001**	3(6.0)	3(1.4)	—	0.054

**Table 4:** Primer used for direct PCR.

Gene	Premier	Nucleotide sequence(5′–3′)	fragment length(bp)
*Tcd*A	NK1	GGACATGGTAAAGATGAATTC	546
	NK2	CCCAATAGAAGATTCAATATTAAGCTT	
*Tcd*B	NK104	GTGTAGCAATGAAAGTCCAAGTTTACGC	204
	NK105	CACTTAGCTCTTTGATTGCTGCACCT	
Triose phosphate isomerase(*tpi*)	tpi-F	AAAGAAGCTACTAAGGGTACAAA	230
	tpi-R	CATAATATTGGGTCTATTCCTAC	
16SrRNA	B	CCGTCAATTCMTTTRAGTTT*	270
	PG-48	CTCTTGAAACTGGGAGACTTG	

^*^M = A or C; R = A or G.
