# Corrigendum: *Clostridium Difficile* Infection Worsen Outcome of Hospitalized Patients with Inflammatory Bowel Disease

**DOI:** 10.1038/srep43975

**Published:** 2017-03-23

**Authors:** Ting Zhang, Qian-Yun Lin, Jia-Xi Fei, Yan Zhang, Min-Yi Lin, Shuang-Hong Jiang, Pu Wang, Ye Chen

Scientific Reports 6: Article number: 2979110.1038/srep29791; published online: 07 15 2016; updated: 03 23 2017

This Article contains errors in the Acknowledgements section:

“This work was supported by the grants from National Natural Science Foundation of China (H0306). The content is solely the responsibility of the authors and does not necessarily represent the official views of the agency above. We thank Yong Sun for his advice and clinical assistance. Supported by National Natural Science Foundation of China (NSFC) (8157041627); The National High Technology Research and Development Program of China (863 Program) (2015AA020701)”.

should read:

“This work was supported by the National Natural Science Foundation of China (NSFC) (81570480), and National High Technology Research and Development Program of China (863 Program) (2015AA020701). The content is solely the responsibility of the authors and does not necessarily represent the official views of the agency above. We thank Yong Sun for his advice and clinical assistance”.

